# Comparison of Simulated Keratometry and Total Refractive Power for Keratoconus According to the Stage of Amsler-Krumeich Classification

**DOI:** 10.1038/s41598-018-31008-1

**Published:** 2018-08-20

**Authors:** Kazutaka Kamiya, Yusuke Kono, Masahide Takahashi, Nobuyuki Shoji

**Affiliations:** 1 School of Allied Health Sciences, Kitasato University, Kitasato, Japan; 2 Department of Ophthalmology, Kitasato University, Kitasato, Japan

## Abstract

This study was aimed to assess the simulated keratometry (Sim K) and the total corneal refractive power (TCRP) in eyes with keratoconus with respect to the Amsler-Krumeich classification. We enrolled 100 eyes of 100 keratoconic patients and 25 age-matched normal eyes. The Sim K and TCRP were measured with a rotating Scheimpflug system (Pentacam HR, Oculus). The differences between Sim K and TCRP in the keratoconus group were significantly larger than those in the control group (p < 0.001). The differences between Sim K and TCRP became larger in the progressive stages of the disease (p = 0.191 for stage 1, p = 0.008 for stage 2, p < 0.001 for stage 3, p < 0.001 for stage 4). We found a significant correlation of Sim K with the differences between Sim K and TCRP in keratoconic patients (r = 0.497, p < 0.001). The differences between Sim K and TCRP for keratoconus were significantly larger than those for normal eyes, and the differences between Sim K and TCRP tended to become larger in the progressive stages of the disease. It is suggested that the Sim K readings overestimate the TCRP, especially in advanced keratoconus, and that this discrepancy is a possible source of a hyperopic refractive error after cataract surgery.

## Introduction

Keratoconus is a progressive noninflammatory disorder characterized by anterior protrusion and thinning of the cornea. The progressive thinning and subsequent bulging of the cornea are often accompanied not only by high myopic astigmatism, but also by irregular astigmatism. It has been demonstrated that the spherical equivalent error depends largely on the cone location, but that the cylindrical error is influenced by the cone location and the shape^1^. Although keratoconic eyes has been shown to develop cataract earlier than non-keratoconic eyes^2,3^, it is still difficult to exactly determine the keratometry and to calculate the intraocular lens (IOL) power in such patients in a clinical setting. Indeed, it has been reported that the predictability of cataract surgery was not very high in keratoconic patients, and that a large amount of hyperopic shift often occurred after cataract surgery, especially in advanced keratoconus, when using the keratometric readings for IOL power calculation^2–5^. However, the etiology of the hyperopic shift still remained unclear in such keratoconic subjects. It has been shown that the ratio between the anterior and posterior corneal curvature is not constant in keratoconic eyes^6^, and that not only the anterior but also the posterior corneal curvature is affected in keratoconic eyes^7^. Indeed, it has been reported that both the anterior and posterior corneal curvatures should be considered to precisely achieve IOL power calculation in cases with posterior keratoconus^8,9^. Considering that the keratometric readings obtained by using a corneal topographer or an autokeratometer are mostly used for IOL power calculation in daily practice, and that these readings are theoretically calculated based on the assumption that the ratio of the anterior and posterior curvatures was constant, even in keratoconic patients, we hypothesize that the keratometric readings may overestimate the actual corneal refractive power, especially in advanced keratoconus. Nevertheless, the differences between the keratometry and the total refractive power have not so far been fully elucidated in eyes with keratoconus, according to the stage of the disease. The simulated keratometry (Sim K) is determined as the average keratometry, calculated by using the standard keratometric index (1.3375) and the radius of anterior corneal curvature, and the total corneal refractive power (TCRP) is determined as the total refractive power, calculated by ray tracing through the anterior and posterior corneal surfaces according to Snell’s law. The detailed analysis of the differences between the Sim K and TCRP in healthy and keratoconic subjects may provide further insights not only on understanding of the etiology of refractive error, but also on the precise IOL power calculation for keratoconic patients with cataract. The goal of the present study is twofold; to retrospectively assess these corneal power differences between healthy and keratoconic subjects, and to compare the Sim K and TCRP, with respect to clinical stage of the disease, in a cohort of keratoconic subjects.

## Results

**Figure 1:**
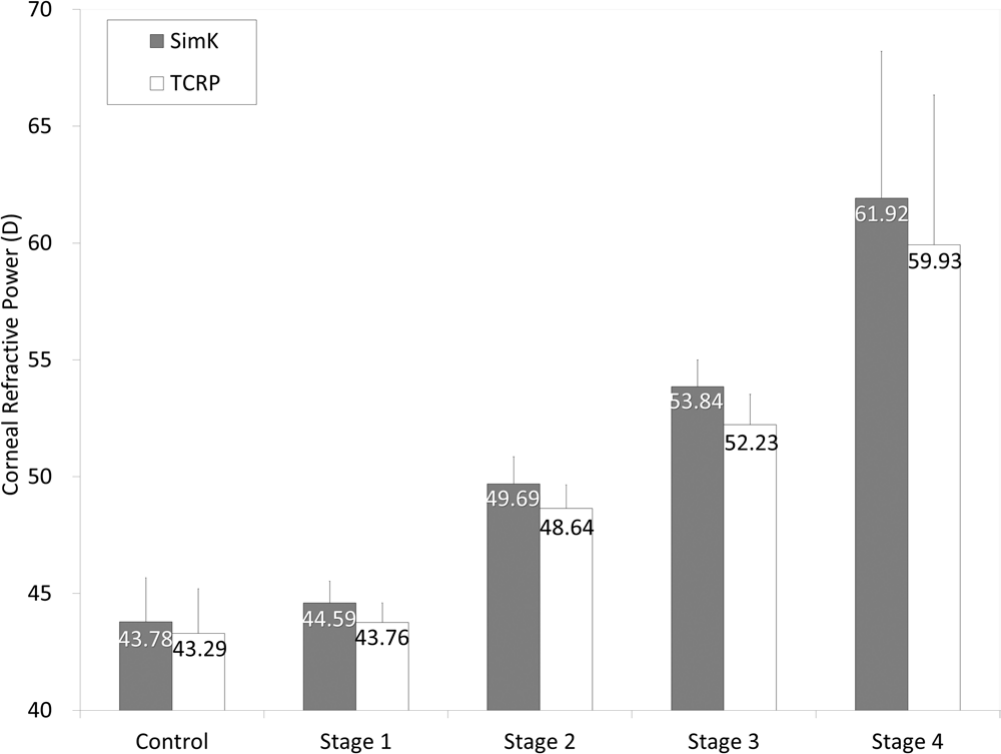
The values of simulated keratometry (Sim K) and total corneal refractive power (TCRP) in the control and keratoconus groups according to the Amsler-Krumeich classification.

**Figure 2:**
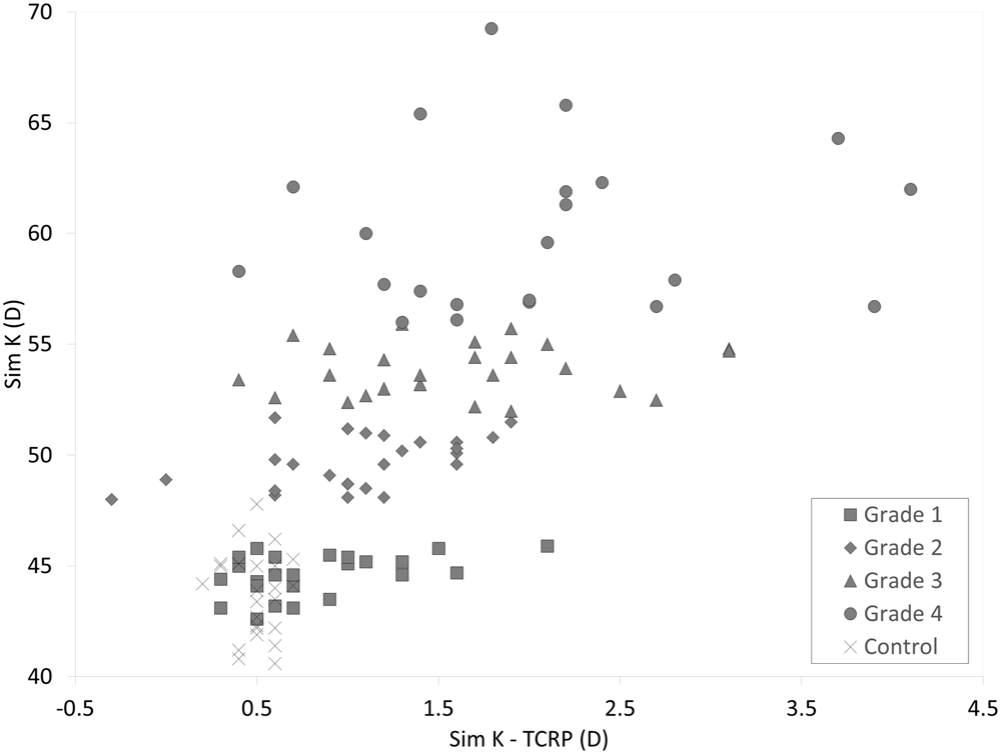
Graphs showing no significant association of simulated keratometry (Sim K) with the differences between Sim K and total corneal refractive power (TCRP) in the control group (Pearson correlation coefficient r = −0.109, p = 0.606), but a significant correlation in the keratoconus group (r = 0.497, p < 0.001).

**Figure 3:**
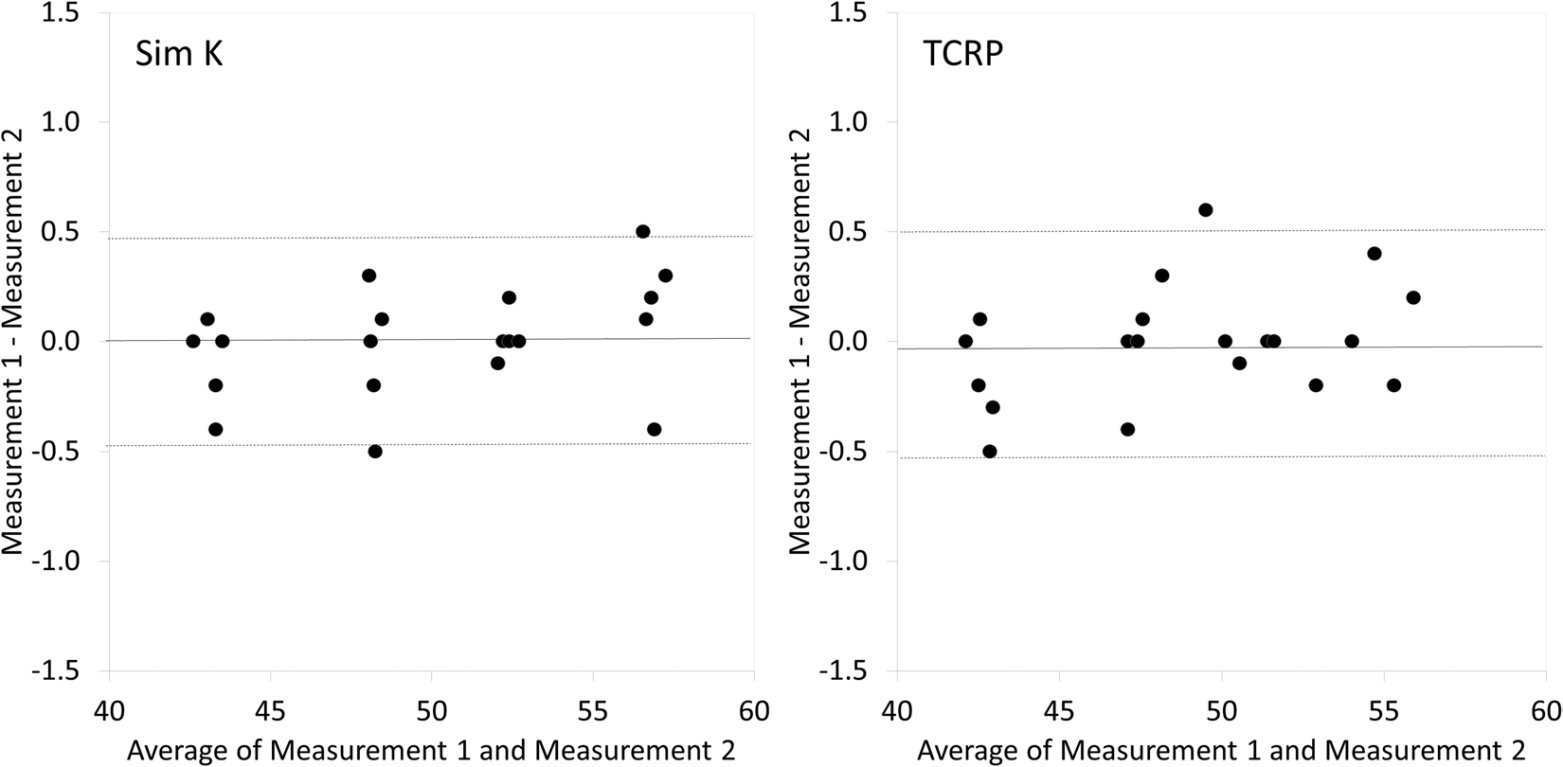
Bland-Altman plots shows the difference between 2 measurements divided by mean of simulated keratometry (Sim K) and total corneal refractive power (TCRP) measurements in eyes with keratoconus. The solid lines represent mean differences between 2 consecutive measurements of corneal refractive power, dotted lines are the upper and lower borders of the 95% LoA (mean difference ± 1.96 multiplied by standard deviation of the mean difference.

The demographics of the study population was summarized in Table 1. The Sim K and TCRP were 52.51 ± 7.15 diopters (D), and 51.14 ± 6.78 D, respectively, in the keratoconus group. The corresponding figures were 43.78 ± 1.89 D and 43.29 ± 1.91 D, in the control group. The values of Sim K were significantly larger than those of TCRP not only in the keratoconus group (p < 0.001, paired t-test) but also in the control group (p < 0.001). The differences between Sim K and TCRP in the keratoconus group were also significantly larger than those in the control group (p < 0.001, Welch’s t-test). We found neither significant correlation of the differences between Sim K and TCRP with age (Pearson correlation coefficient r = −0.008, p = 0.936) in the keratoconus group, nor significant correlation (r = 0.102, p = 0.629) in the control group. We also found no significant differences between Sim K and TCRP between male and female, not only in the keratoconic group (p = 0.692, Welch’s t-test), but also in the control group (p = 0.833). The values of Sim K and TCRP in the control and keratoconus groups according to the stage of the disease were shown in Fig. 1. The variance of the data was statistically significant (p < 0.001, ANOVA). A multiple comparison showed no significant differences between Sim K and TCRP of the control group with the stage 1 keratoconus group, but significant differences with the stage 2 to 4 keratoconus groups (Dunnett test, p = 0.191 for stage 1, p = 0.008 for stage 2, p < 0.001 for stage 3, p < 0.001 for stage 4). Although we found no significant differences between Sim K and TCRP between the control and grade 1 keratoconus groups, the differences became larger with the progressive stages of the disease. We found no significant correlation of Sim K with the differences between Sim K and TCRP in the control group (Pearson correlation coefficient r = −0.109, p = 0.606), but a significant correlation in the keratoconus group (r = 0.497, p < 0.001) (Fig. 2). Bland-Altman plots show that the mean difference between the two measurements with this instrument (±95% limits of agreement [LoA]) was 0.00 ± 0.25 D (−0.49 to 0.49 D) for Sim K, and −0.01 ± 0.26 D (−0.52 to 0.50 D) for TCRP (Fig. 3).

**Table 1:** Demographics of the study population in the keratoconus and control groups.

Characteristic	Keratoconus group	Control group	P-value
Number of subjects	100	25	
Age	36.9 ± 12.0 years (95%CI, 13.4 to 60.5 years)	35.0 ± 7.3 years (95%CI, 22.0 to 48.0 years)	0.143
Sex	73 men and 27 women	15 men and 10 women	0.878
Manifest spherical equivalent	−5.77 ± 4.66 D (95%CI, −14.91 to 3.36 D)	−5.69 ± 2.56 D (95%CI, −10.70 to −0.68 D)	0.547
Sim K	52.51 ± 7.15 D (95%CI, 38.50 to 66.52 D)	43.78 ± 1.89 D (95%CI, 40.08 to 47.49 D)	<0.001
TCRP	51.14 ± 6.78 D (95%CI, 37.86 to 64.42 D)	43.29 ± 1.91 D (95%CI, 39.55 to 47.03 D)	<0.001
Δ Sim K – TCRP	1.37 ± 0.83 D (95%CI, −0.25 to 2.99 D)	0.50 ± 0.12 D (95%CI, 0.25 to 0.74 D)	<0.001

CI = confidence interval, D = diopter, Sim K = simulated keratometry, TCRP = total corneal refractive power.

## Discussion

In the present study, our results revealed that Sim K and TCRP in the keratoconus group were significantly larger than those in the control group, and that the differences between Sim K and TCRP in the keratoconus group were significantly larger than those in the control group. We found no significant association of these differences with age or sex, not only in the keratoconic group, but also in the control group, indicating that these keratometric differences were not significantly affected by age or sex in this study population. Our results also showed that the differences between Sim K and TCRP tended to become larger in the progressive stages of the disease. It is suggested that the Sim K readings overestimate the TCRP, and that this tendency is more prominent especially in eyes with the more progressive staged keratoconus. The overestimation of the corneal refractive power may lead to the selection of the lower IOL power, resulting in a hyperopic refractive error in IOL-implanted eyes with keratoconus. Actually, Leccisotti *et al*. stated that the IOL exchange due to imprecise IOL power occurred in 32% after refractive lens exchange for keratoconus^2^. Watson *et al*. mentioned that the use of actual keratometric readings can result in a large hyperopic error for severe keratoconus^4^. Park *et al*. found that a hyperopic shift was noted since localized corneal posterior elevation is not reflected in conventional IOL power calculation for posterior keratoconus^8^. It has been reported that the real corneal power that takes both the anterior and posterior corneal curvatures into consideration should be applied for IOL power calculation in cases with posterior keratoconus^9,10^. Camps *et al*. demonstrated that the use of a single value of the keratometric index for the calculation of the total corneal power in keratoconus has been shown to be imprecise, leading to inaccuracies in the detection and classification of this corneal condition^11^. Therefore, we believe that our findings was simple, but helpful, for understanding the etiology of a hyperopic shift after cataract surgery, when the keratometric readings were applied, and for adjusting IOL power calculation for keratoconus in daily practice. We should be aware that there is a need for optimizing IOL power when we calculated IOL power using the conventional keratometric readings, and that the TCRP, instead of the Sim K, may be useful for IOL power calculation, especially for advanced keratoconus.

To date, there have been several studies on detailed analysis of the corneal refractive power of the posterior surface in eyes with keratoconus^7,12,13^. Tomidokoro *et al*. showed that both anterior and posterior curvatures were influenced in eyes with keratoconus as well as in keratoconus-suspect eyes, and that these changes are observed from the early stage of the disease^7^. Piñero *et al*. stated that the association between anterior and posterior corneal curvatures was lower in keratoconic eyes^12^. Reddy *et al*. demonstrated that total corneal power, anterior curvature, posterior curvature, pachymetry, and corneal aberration data were useful for differentiating keratoconus and early keratoconus eyes from normal eyes^13^. As far as we can ascertain, this is the first study to evaluate the differences between Sim K and TCRP for keratoconus according to the clinical stage of this disorder. This discrepancy may contribute to a hyperopic shift after cataract surgery, especially in eyes with advanced keratoconus. We are currently conducting a new study on the relationship of its discrepancy with actual refractive error after cataract surgery for keratoconus.

It is clinically essential to validate the repeatability of the corneal refractive power measurements with the device. As shown in Fig. 3, we confirmed the good repeatability of the Sim K and TCRP measurements, as evidenced by the narrow 95% LoA, in the present study. Furthermore, it has been shown that the Scheimpflug system has an excellent repeatability of the corneal curvature measurements even in eyes with keratoconus^14^. Hence, we believe that the instrument offers clinically reasonable repeatability even in the assessment of corneal refractive power for keratoconus.

There are at least two limitations to this study. One is that it was conducted in a retrospective fashion. A randomized, controlled study would be ideal for confirming the authenticity of our results. Another limitation is that we determined the Sim K and TCRP on the 3.0-mm ring only using the Scheimpflug imaging system, because this measurement is considered to be simple and easy to quantitatively grasp corneal refractive power in keratoconic patients. However, anterior segment optical coherence tomographer may have advantages over the Scheimpflug system in terms of accuracy and reproducibility, especially in keratoconic eyes having corneal opacity^15^.

In summary, our findings support the view that the differences between Sim K and TCRP for keratoconus were significantly larger than those for normal eyes, and that the differences between Sim K and TCRP tended to become larger with the progressive stages of the disease. Based on our findings, it is indicated that the Sim K readings may overestimate the TCRP, especially in advanced keratoconus. This overestimation of the corneal refractive power may lead to the selection of the lower IOL power, and subsequently result in a hyperopic shift after cataract surgery, especially in eyes with the progressive stages of the disease, when the keratometric readings were utilized for the IOL power calculation.

## Methods

### Study Population

The study protocol was registered with the University Hospital Medical Information Network Clinical Trial Registry (000034266). This retrospective study comprised 100 eyes of 100 keratoconic patients (73 men and 27 women, mean age ± standard deviation (SD): 36.9 ± 12.0 years) with good quality scans of corneal tomography measured with a rotating Scheimpflug imaging instrument (Pentacam HR^TM^, Oculus, Wetzlar, Germany) as the study group, and age-matched 25 normal eyes as the control group, at Kitasato University Hospital between January 2016 and December 2017. We randomly enrolled only one eye per subject for statistical analysis. Some of the subjects were those in our preceding reports on corneal height information for keratoconus^16,17^. The sample size in the present study offered 85.0% statistical power at the 5% level in order to detect a 1-D difference in the corneal refractive power, when the SD of the mean difference was 1.6 D. Diagnosis of keratoconus was performed by one experienced clinician (K.K.) with evident findings characteristic of keratoconus (e.g., corneal topography with asymmetric bow-tie pattern with or without skewed axes), and at least one keratoconus sign (e.g., stromal thinning, conical protrusion of the cornea at the apex, Fleischer ring, Vogt striae, or anterior stromal scar) on slit-lamp examination^18^. Eyes with pellucid marginal degeneration, other corneal diseases, and previous ocular trauma or surgery, were excluded from the study. We divided the study group into 4 (Grade 1 to 4) keratoconus subgroups, according to the Amsler-Krumeich classification, based on astigmatism, corneal power, corneal transparency, and corneal thickness^19^, obtained using the rotating Scheimpflug imaging instrument and slit-lamp biomicroscopy. The patients were recruited in a continuous cohort, until the number of eyes at each keratoconus stage has been reached to 25 eyes. The patients who wore rigid gas permeable and soft contact lenses were asked to stop wearing them for 3 and 2 weeks before this evaluation, respectively, in order to exclude the effect of wearing contact lenses. This retrospective review of the data was approved by the Institutional Review Board at Kitasato University and followed the tenets of the Declaration of Helsinki. Our Institutional Review Board waived the requirement for informed consent for this retrospective study.

### Assessment of Simulated Keratometry and Total Corneal Refractive Power

The values of Sim K and TCRP on the central 15° ring (equal to the 3.0-mm ring) around the corneal apex were automatically measured with the Scheimpflug imaging system (Pentacam HR, software version 1.20) by experienced optometrists. After achieving perfect alignment, we took 25 Scheimpflug images within 2 seconds by this instrument. We checked image quality for each eye, and only one examination with a high quality factor was documented. In order to evaluate the repeatability of the measurements, the measurements of Sim K and TCRP were additionally made at the same time of day on two consecutive days in 20 keratoconic eyes. We evaluated the repeatability of the two measurements using Bland-Altman plots, as described previously^20^.

### Statistical Analysis

We conducted statistical analyses by using a commercially available statistical software (Bellcurve for Excel, Social Survey Research Information Co, Ltd., Tokyo, Japan). The paired t-test was used to assess the differences of the two variables in each group. The Welch’s t-test was used to compare the data between the keratoconic and control groups. The Pearson correlation coefficient was calculated to assess the relationships of the two variables. One-way analysis of variance (ANOVA) was used to assess the differences between Sim K and TCRP in each keratoconus and control groups, with the Dunnett test being employed for multiple comparison. The results are expressed as mean ± SD, and a value of p < 0.05 was considered statistically significant.

## Data Availability

The datasets generated during and/or analysed during the current study are available from the corresponding author on reasonable request.
